# Obesity and long bone fractures in children. Systematic review

**DOI:** 10.1007/s00402-026-06221-7

**Published:** 2026-03-10

**Authors:** Ahmed Aly, Tarek Aly

**Affiliations:** 1 InternalMedicine Department, Tanta University Hospital, Tanta, Egypt; 2 Department oforthopedicsand traumatology, Facultyof Medicine, Tanta University, Tanta, Egypt

**Keywords:** Childhood obesity, Long bone fractures, Pediatric orthopedics, Fracture patterns, Surgical outcomes, Bone health.

## Abstract

**Background:**

Childhood obesity is a pressing global health issue with potential implications for musculoskeletal injury risk and recovery. Although the influence of obesity on bone metabolism is acknowledged, its specific connection to the incidence, patterns, and treatment results of long bone fractures in the pediatric demographic requires further clarification.

**Purpose:**

This systematic review aims to evaluate the existing literature on the relationship between obesity and long bone fractures in children and adolescents, with a focus on fracture risk, anatomical distribution, management approaches, and clinical outcomes.

**Methods:**

We conducted a systematic literature search of PubMed, MEDLINE, Cochrane Library, and Google Scholar for studies published from January 2000 to March 2025. Search terms included “childhood obesity”, “bone health”, and “long bone fractures”. We included English-language original research that analyzed the relationship between pediatric obesity (BMI ≥ 95th percentile) and long bone fractures. Data on study characteristics, fracture patterns, treatments, and outcomes were extracted.

**Results:**

Out of 2,152 articles screened, 14 met the inclusion criteria. Children who were overweight or obese had higher odds of lower extremity fractures, particularly of the tibia and femur (odds ratios 1.5–3.3). Obesity was linked to more complex fracture patterns, including physical involvement and displacement, and a higher failure rate of nonsurgical management, especially for forearm and supracondylar fractures. Operative complications, such as varus deformity and pin-related issues, were more frequent in obese patients. However, no consistent differences were observed between obese and non-obese groups regarding mortality and long-term functional outcomes.

**Conclusions:**

Pediatric obesity significantly elevates the risk and complexity of long bone fractures and complicates both nonoperative and surgical management. Acknowledging these challenges is crucial for optimizing treatment and preventing adverse outcomes. Further multicenter prospective studies are needed to clarify the underlying biomechanical and metabolic mechanisms and to guide the development of individualized management protocols.

**Level of evidence:**

Systematic Review, Level III.

## Introduction

The global prevalence of obesity continues to rise, including among children and adolescents. Since the 1970 s, obesity rates in the 6 to 19-year-old pediatric demographic have tripled [1, 2]. The Centers for Disease Control categorizes pediatric body weight as underweight (< 5th percentile), normal or healthy weight (5th to < 85th percentile), overweight (85th to < 95th percentile), and obese (≥ 95th percentile).

Obesity is a known risk factor for specific orthopedic conditions like slipped capital femoral epiphysis and Blount disease. A growing body of evidence also suggests that obesity independently increases the risk of fracture-related complications and adversely affects surgical management, in addition to inducing physiological changes in the growing skeleton compared to healthy-weight individuals [3]. While some studies indicate that injured obese children present with similar injury severity and mortality rates as their non-overweight counterparts [4–7], they appear to sustain different anatomical patterns of injury.

Nevertheless, the current literature on post-traumatic complications in obese children is largely composed of single-institution studies, which limits the generalizability of their findings. Previous research has also yielded conflicting results regarding the impact of obesity on complications and the utilization of hospital resources, such as ICU needs [8–10].

Grasping the impact of obesity-related pathways on bone is vital for understanding its broader implications beyond the more commonly recognized comorbidities. A key driver of these comorbidities is insulin resistance [11]. The limited capacity of adipose tissue to expand leads to an overproduction of free fatty acids, which accumulate in the liver and muscle. This buildup impairs insulin signaling by promoting the formation of diacylglycerol and ceramides. This process, combined with dysregulated lipolysis in adipose tissue, worsens insulin resistance, paving the way for conditions like fatty liver disease, type 2 diabetes, and metabolic syndrome. Concurrently, hyperinsulinemia alters the differentiation of mesenchymal stem cells, promoting adipogenesis over osteoblastogenesis, thereby reducing bone formation [12].

The relationship between obesity and bone health is also shaped by mechanical and inflammatory factors. On one hand, greater body weight increases mechanical loading, which can stimulate cortical bone formation. On the other hand, the chronic low-grade inflammation associated with obesity counteracts this benefit, negatively impacting bone quality, particularly in areas rich in trabecular bone [13]. This inflammatory state is marked by elevated levels of pro-inflammatory cytokines like TNF-α and IL-6, which activate the receptor activator of nuclear factor kappa-B ligand (RANKL) [14]. RANKL, produced mainly by osteoblastic lineage cells and immune cells during inflammation, promotes osteoclastogenesis by binding to its receptor, RANK, on osteoclast precursors. Osteoprotegerin (OPG), a decoy receptor for RANKL, regulates this pathway and is crucial for bone protection. Disruption of the RANKL/RANK/OPG axis has been identified as a factor contributing to bone changes in various congenital and acquired pediatric conditions, including obesity [15–17].

This review systematically assesses whether childhood obesity increases the risk, complexity, and adverse outcomes of long bone fractures.

## Materials and methods

### Study design

This systematic review was conducted following the Preferred Reporting Items for Systematic Reviews and Meta-Analyses (PRISMA 2020) guidelines. The protocol was designed to identify and synthesize available evidence on the association between obesity and long bone fractures in children and adolescents. Human Ethics and Consent to Participate declarations: not applicable.

### Eligibility criteria

Studies were included if they met the following criteria:

1. Population: Children and adolescents aged 0–19 years.
2. Exposure: Overweight or obesity defined by body mass index (BMI) ≥85th percentile (overweight) or ≥ 95th percentile (obese) for age and sex.
3. Comparator: Normal or underweight pediatric populations.
4. Outcomes: Incidence, pattern, or outcomes of long bone fractures (humerus, radius/ulna, femur, or tibia/fibula).
5. Study Design: Observational studies (retrospective, prospective cohort, or case–control) and randomized or quasi-experimental trials.
6. Language: English.
7. Publication Type: Peer-reviewed original research articles published between January 2000 and March 2025.

Exclusion criteria included: non-human studies, case reports, conference abstracts, reviews, studies evaluating fractures in non-long bones or metabolic bone diseases unrelated to obesity, and papers lacking BMI data or stratification by weight categories.

### Information sources and search strategy

A comprehensive search was conducted across PubMed, MEDLINE, Cochrane Library, and Google Scholar databases, with the final search performed on March 15, 2025. The search string was adapted for each database: (“childhood obesity” OR “pediatric obesity” OR “overweight children”) AND (“long bone fractures” OR “upper extremity fractures” OR “lower extremity fractures” OR “femur fracture” OR “tibia fracture” OR “humerus fracture” OR “forearm fracture”) AND (“children” OR “adolescents”). Boolean operators “AND” and “OR” were applied as shown. Duplicates were removed using EndNote X9 software.

### Study selection

**Fig. 1:**
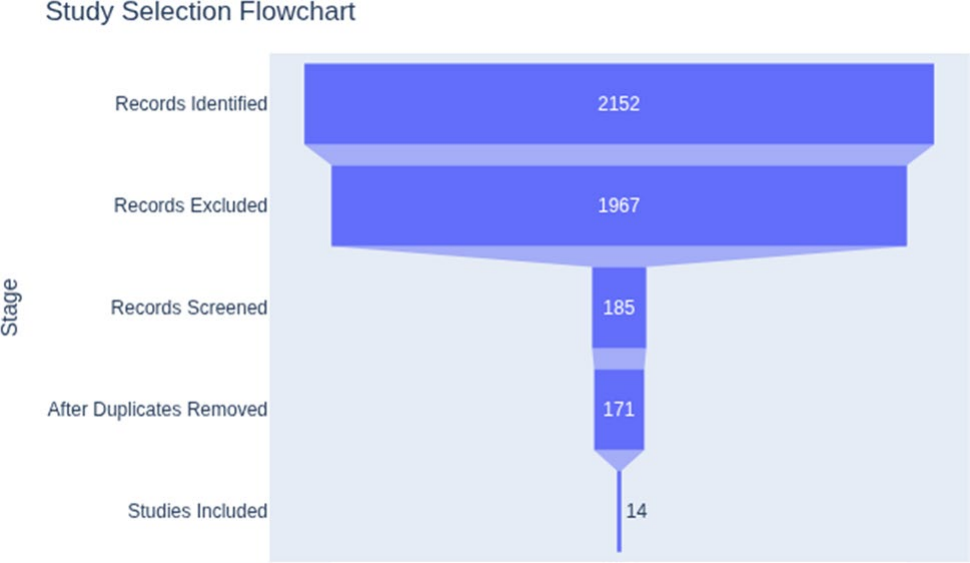

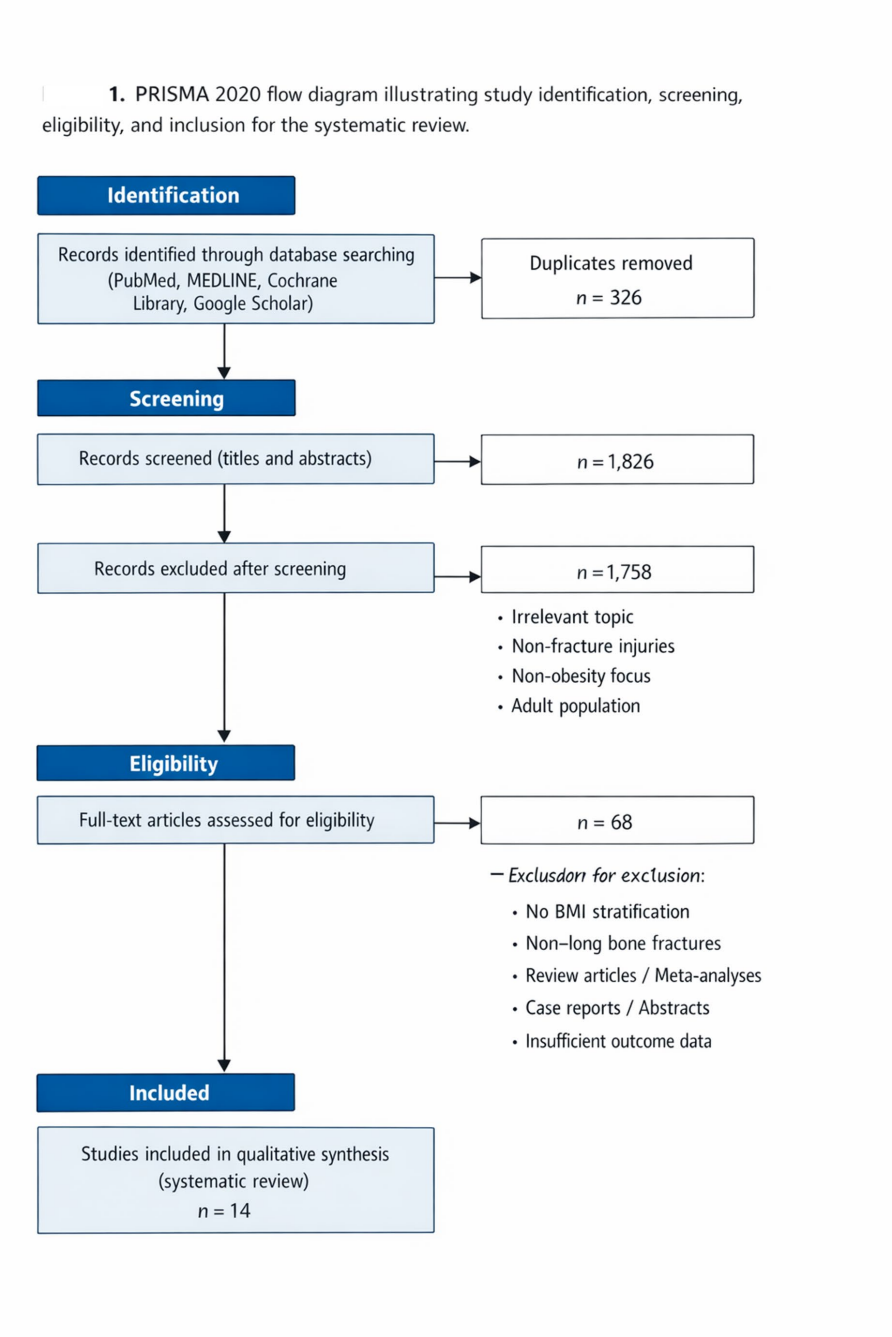
PRISMA flowchart of study selection

Two reviewers independently screened titles and abstracts for relevance. The full texts of potentially eligible studies were reviewed to confirm inclusion. Disagreements were resolved through discussion and consensus. The selection process is summarized in a PRISMA flow diagram (Fig. 1).

### Data extraction

A standardized data extraction form was used to collect the following variables from each study: author(s), year, country, study design, sample size, age range, sex distribution, definition of obesity, method of BMI classification, fracture site(s) and type(s), main outcomes (incidence, complications, management approach, prognosis), and reported effect measures (odds ratios, relative risks, p-values).

### Quality assessment

The methodological quality of the included studies was appraised using the Newcastle–Ottawa Scale (NOS) for observational studies. Each study was evaluated on selection, comparability, and outcome/exposure assessment, with a maximum score of 9 points. Studies scoring ≥ 7 were considered high quality, 5–6 moderate quality, and ≤ 4 low quality.

### Data synthesis

Due to heterogeneity in study design, outcome definitions, and data reporting, a meta-analysis was not feasible. A qualitative synthesis was performed, summarizing findings according to fracture site, obesity classification, and clinical outcomes.

## Results

### Study characteristics

The screening of 2,152 papers yielded 14 studies that met the inclusion criteria for evaluating the relationship between childhood obesity and long bone fractures. These studies, conducted between 2012 and 2022 in various geographical settings, predominantly used retrospective or cross-sectional designs. Sample sizes varied widely, from 57 to over 900,000 pediatric patients aged 2–19 years (Table 1).

**Table 1:**
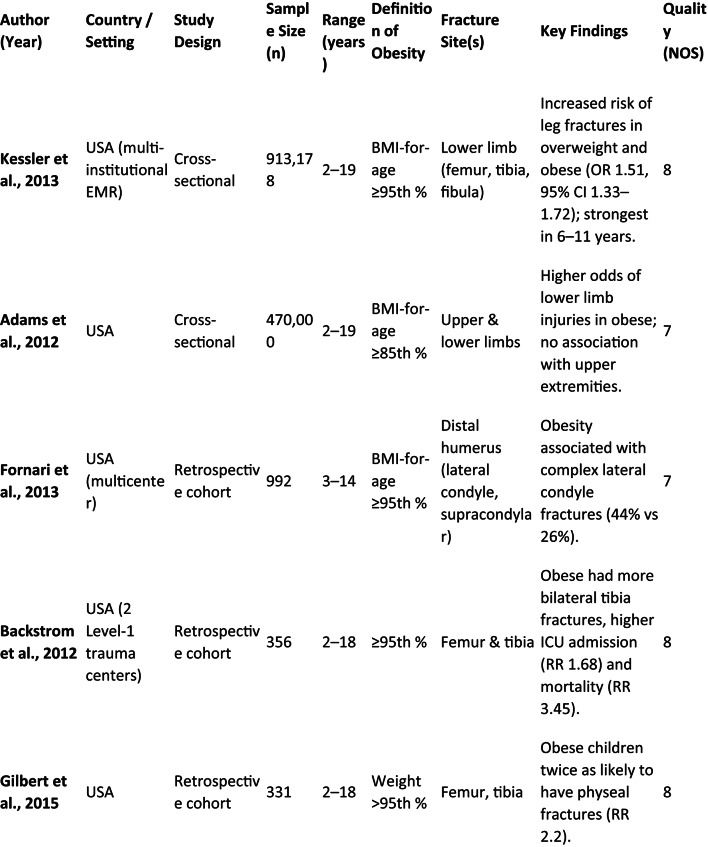
Summary of studies evaluating the relationship between pediatric obesity and long bone fractures (2000–2005)

1. Upper Limb Fractures:

While Fornari et al. (2013) in a review of 992 distal humeral fractures reported a higher prevalence of obesity among children sustaining lateral condyle fractures (37%) compared with supracondylar fractures (19%), Mitchelson et al. (2013) in their assessment of 382 pediatric supracondylar fractures found no significant differences in fracture severity or complication rates within supracondylar fractures across BMI categories [18]. Furthermore, among lateral condyle fractures, the more severe as Salter-Harris Type III patterns (displaced lateral condyle fractures with articular involvement) were more prevalent in obese children (44%) compared to Types I-II (27%−26%) [19].

However, other studies pointed to a different conclusion. Seeley et al. (2014) demonstrated that in 354 children with supracondylar humeral fractures, obesity was strongly correlated with more displaced fracture patterns (A higher proportion of Gartland type III–IV supracondylar fractures characterized by complete displacement and multidirectional instability.) (OR = 9.19, *p* < 0.001) and higher rates of both preoperative (OR = 2.69) and postoperative nerve palsies (OR = 7.69). Notably, these complex fractures in obese patients often resulted from low-energy mechanisms [20].

The increased need for open reduction in obese children may not solely reflect greater fracture displacement but may also be attributable to technical difficulties during closed reduction caused by a thicker soft-tissue envelope, impaired fluoroscopic visualization, and limited ability to apply corrective forces.

The challenges extended to surgical outcomes. Chang et al. (2015) compared 107 children with type III supracondylar fractures and found that obese patients were more prone to developing varus angulation (*p* = 0.017) and pin-related complications (*p* = 0.013) [21]. Çabuk et al. (2016) also observed that among 57 children with supracondylar fractures, a higher BMI was significantly associated with the need for open reduction and internal fixation over closed reduction (*p* < 0.01), suggesting more severe initial displacement [22].

Regarding fracture incidence, Aldo et al. (2015) reported that obese children were twice as likely to sustain upper-limb fractures (RR = 1.97) and more frequently required manipulation under anesthesia [23]. This increased susceptibility was supported by Akshatha et al. (2020), who found a higher prevalence of forearm fractures in obese children [24].

Conservative management also proved less effective in this population. Okoroafor et al. (2017) studied 129 both-bone forearm fractures and found that non-surgical management failed in 34% of overweight/obese children, compared to 18% in the normal-weight group (*p* < 0.05). Over half of these failures subsequently required surgery [25]. Nhan et al. (2021) further highlighted that among 608 patients with isolated upper-limb fractures, overweight and obese children sustained more complete fractures (65% vs. 55%) and more physeal injuries (37% vs. 23%; *p* = 0.007) [26].

2. Lower Limb Fractures:

The association between obesity and lower limb fractures was particularly strong. A large cross-sectional analysis by Adams et al. (2012) of 470,000 pediatric cases showed that overweight (OR = 1.18), moderately obese (OR = 1.24), and extremely obese (OR = 1.34) children had significantly higher odds of lower-limb injuries, with no consistent association found for upper-limb trauma [27]. Kessler et al. (2013), in an even larger analysis of 913,178 children, confirmed an increased risk of leg fractures in overweight and obese patients (OR = 1.51), especially in the 6–11-year-old age group [28].

The pattern of lower limb injuries also differed. Backstrom et al. (2012) noted that in 356 patients with femur and tibia fractures, obese patients were older, had more bilateral tibial fractures, and required operative management for femur fractures more frequently. After adjusting for age, these children also faced higher risks of ICU admission (RR = 1.68) and in-hospital mortality (RR = 3.45) [29].

Physeal involvement was a recurring theme. The increased incidence of physeal injuries in obese children predominantly involved Salter–Harris type II fractures of the distal femur and proximal tibia, with some studies reporting higher rates of distal femoral physeal separation. Gilbert et al. (2015) reported that obese children with femoral or tibial fractures were twice as likely to sustain physeal injuries (RR = 2.20), with a greater risk for femur fractures (RR = 3.25) than for tibia (RR = 1.58) [30]. McGregor et al. (2022) also found that among 215 tibial fractures, overweight and obese children had a much higher rate of physeal fractures (54% vs. 29%) and more distal-third fractures, often from low-energy mechanisms [31].

Although operative fixation is standard for femoral shaft fractures in children aged 6–11 years, obese patients were more likely to require more invasive or rigid fixation methods and experienced higher complication rates.

The increased mechanical stress in heavier children complicated treatment. Andreacchio et al. (2019) examined 679 children with femur and tibia fractures treated by elastic stable intramedullary nailing (ESIN) and found that those weighing > 50 kg had a significantly higher complication rate (27.9%), which was especially pronounced in femoral fractures (51.7%) [32].

## Summary of findings

Thirteen of the fourteen included studies indicated that obesity elevates the risk, severity, and complication rates of long bone fractures in children (Table 2). Consistent findings across these studies include:

**Table 2:**
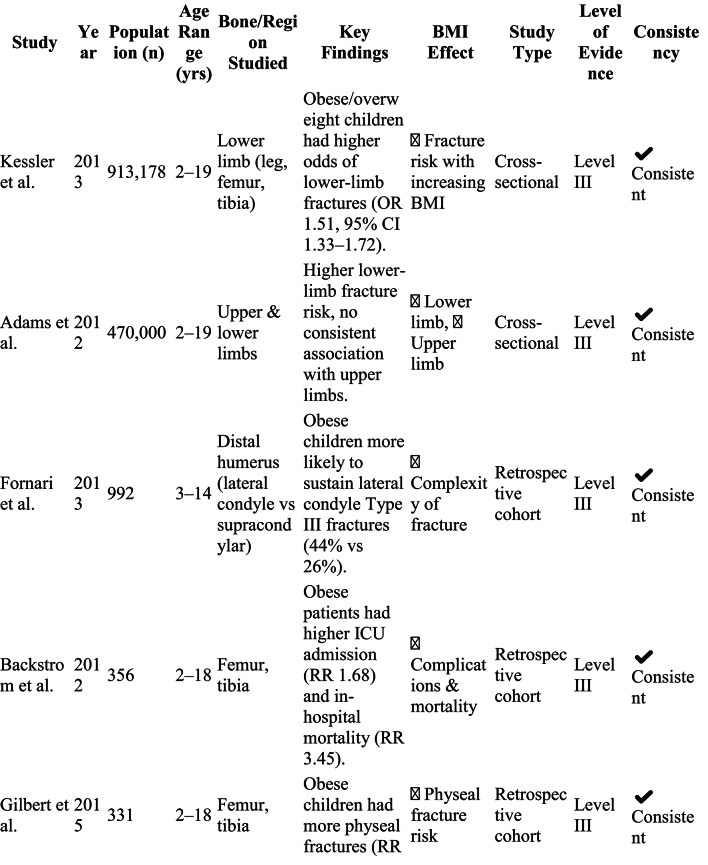
Summary of included studies on the effect of obesity on long bone fractures in children

**Fig. 2:**
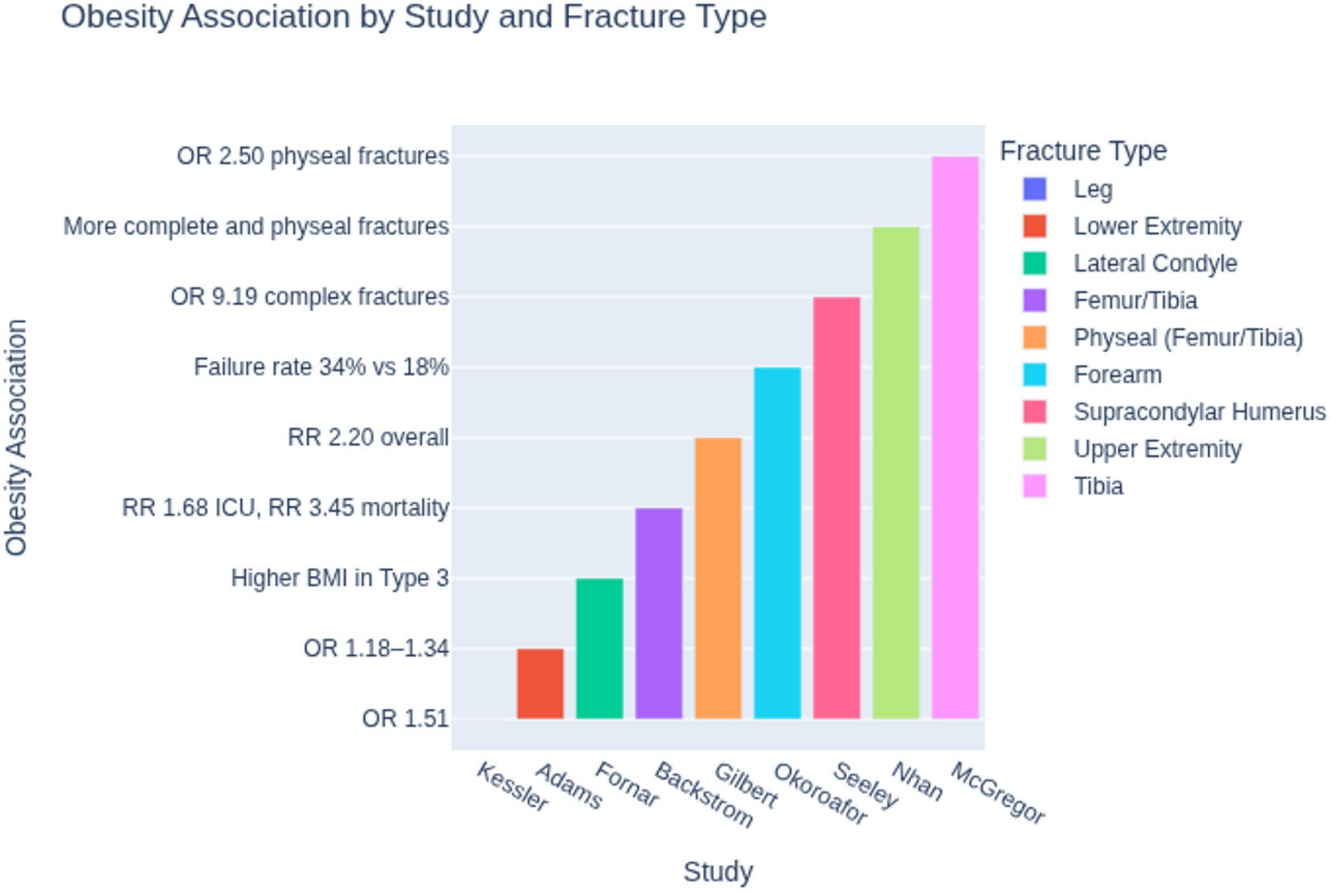
Obesity association by fracture type

**Fig. 3:**
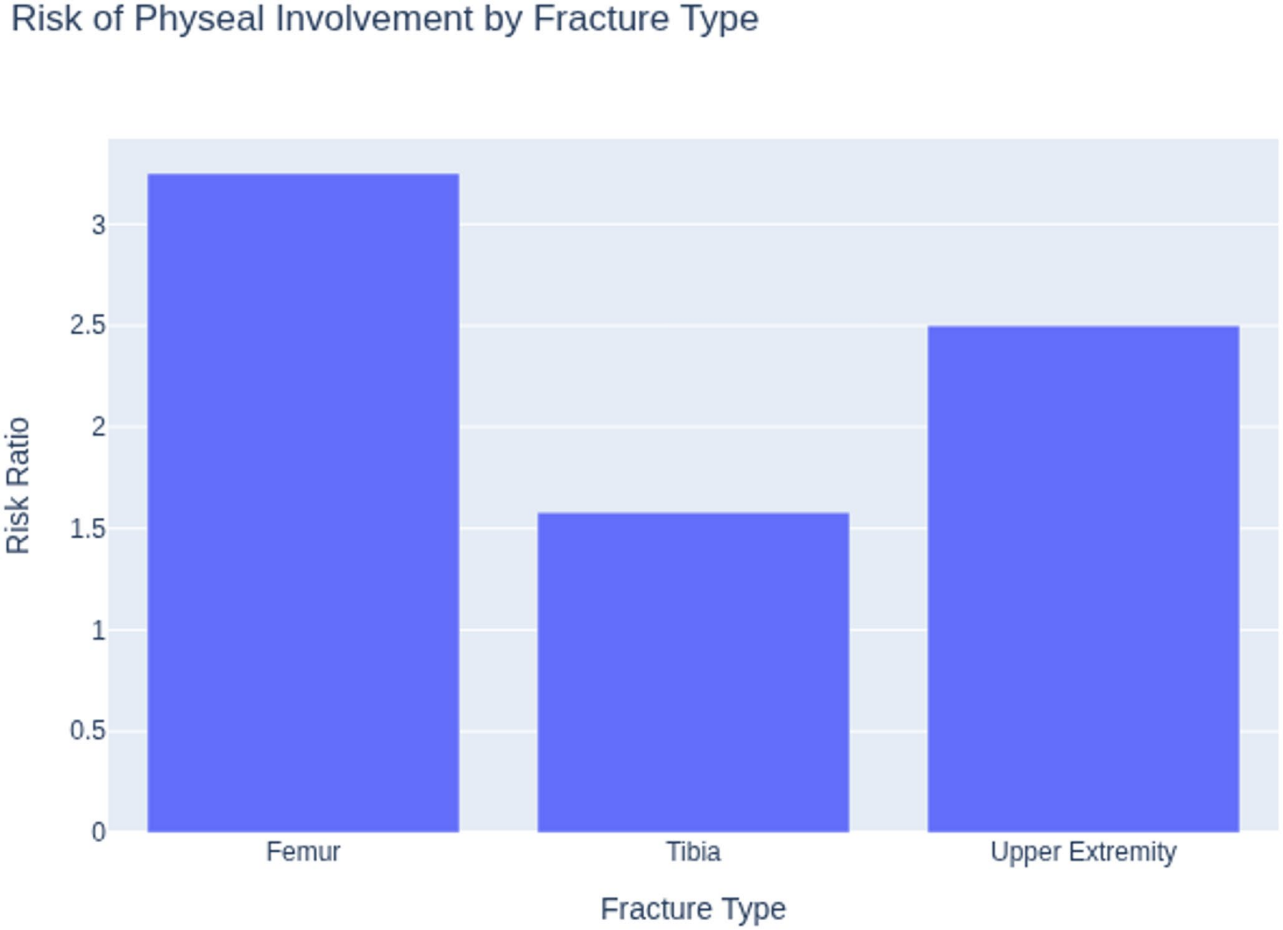
Risk of physeal involvement by fracture type

**Fig. 4:**
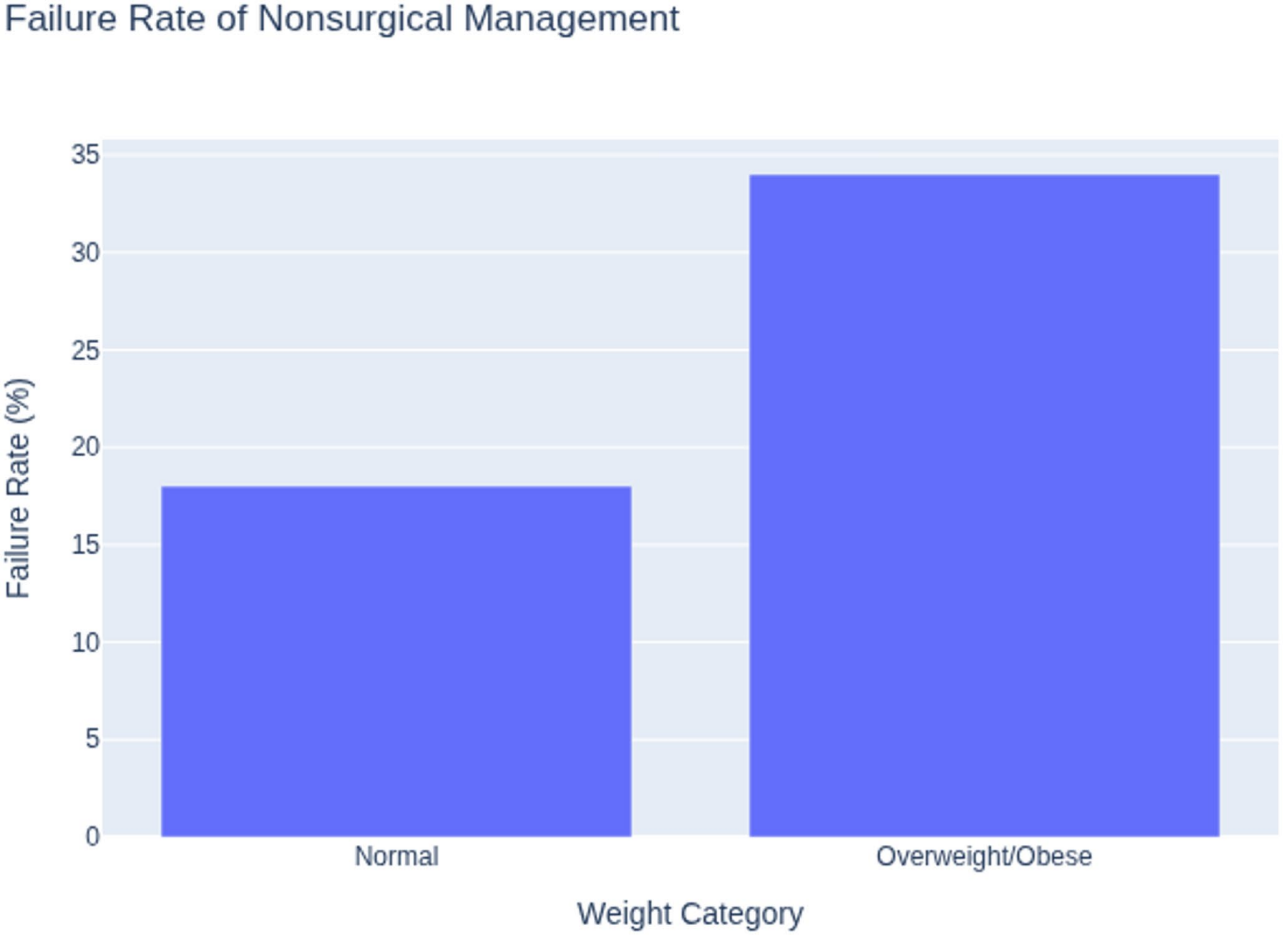
Failure rate of conservative management in both-bone forearm fractures

**Fig. 5:**
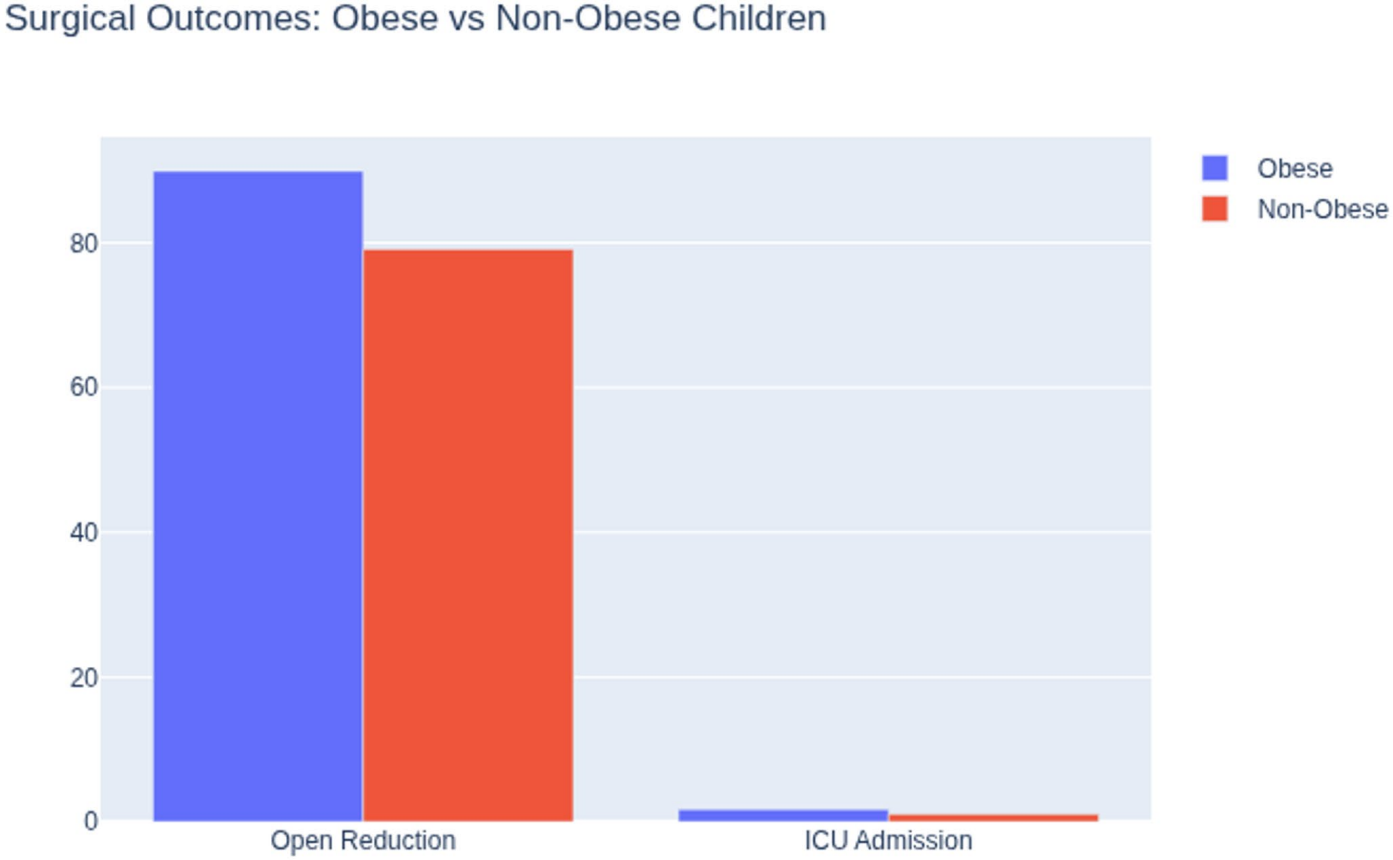
Surgical outcomes in obese vs. non-obese children

- A higher incidence of lower-limb fractures, particularly of the tibia and femoral shaft (Fig. 2).

- A greater frequency of physeal and complete fractures in both the upper and lower extremities (Fig. 3).

- Increased technical difficulty and higher complication rates during operative fixation.
- Reduced success rates for conservative management, especially for both-bone forearm fractures (Figs. 4 and 5).

## Discussion

### Epidemiologic evidence

This systematic review demonstrates that childhood obesity is consistently associated with altered fracture patterns, increased physeal involvement, and higher complication rates following long bone fractures. Within the studies reviewed, the prevalence of obesity among pediatric trauma patients varied from 19% to 46%, mirroring the worldwide trend of rising BMI in children [33, 34]. Large, population-based studies consistently demonstrated that overweight and obese children experience a greater number of long bone fractures than their normal-weight peers. Research by Kessler et al. and Adams et al. specifically identified increased odds of lower-extremity fractures (OR 1.5–3.3) in these children, with a pronounced effect in the 6 to 11-year-old range [27, 28]. While earlier trauma literature suggested comparable overall injury severity between weight groups [4–7], the current synthesis indicates that obesity fundamentally alters the *fracture pattern* and the *energy transfer* during injury, leading to a distinct orthopedic profile, even when factors like age and injury mechanism are accounted for.

### Fracture pattern variations

The reviewed literature consistently documented distinct anatomical fracture patterns in obese children. A clear predominance of lower-limb injuries was observed, with femoral and tibial fractures constituting the majority [27–31]. A key finding was the increased vulnerability of the physis. Studies by Nhan, Gilbert, and McGregor collectively reported that obese children were approximately twice as likely to suffer physeal fractures compared to their non-obese peers [26, 30, 31]. In the upper limb, obesity was correlated with more complex distal humeral fractures, especially lateral condyle and severely displaced supracondylar types [18–22]. These observations confirm a shift from the incomplete fractures (e.g., buckle or greenstick) typical of healthy-weight children toward complete, displaced, or articular fractures in those with obesity, pointing to a combination of altered biomechanics and compromised bone quality.

### Treatment complications and outcomes

Obesity negatively impacts both conservative and surgical fracture management. The failure rate of nonsurgical treatment was significantly higher in overweight and obese children with both-bone forearm fractures—34% versus 18% in normal-weight patients [25]. The higher failure rate of conservative management in obese children has been attributed to difficulties in achieving and maintaining adequate cast molding, increased mechanical loading across the fracture site, and loss of reduction due to soft-tissue interposition [25]. Surgical fixation also presents considerable challenges. Obese children with type III supracondylar humeral fractures faced greater risks of varus deformity, pin-site infection, and loss of reduction [20, 21]. The increased incidence of varus malalignment in obese children may result from suboptimal reduction, limited stability of standard pin configurations under higher mechanical loads, and difficulty achieving accurate coronal alignment intraoperatively. Several authors advocate the use of larger-diameter Kirschner wires, additional lateral pins, or cross-pin configurations, as well as closer postoperative radiographic surveillance in obese patients [20–22].

The likelihood of requiring open reduction was greater in high-BMI patients [22], and complication rates nearly doubled in children weighing > 50 kg after elastic stable intramedullary nailing of femur or tibia fractures [32]. Elastic stable intramedullary nailing is generally not recommended in children weighing more than 50 kg due to higher rates of mechanical failure, a recommendation supported by regulatory and biomechanical considerations. Furthermore, Backstrom et al. reported increased ICU admissions (RR 1.68) and in-hospital mortality (RR 3.45) in obese pediatric trauma patients [29]. Together, these results underscore that obesity complicates fracture care by increasing both the technical difficulty of procedures and the postoperative complication rate. It should be to noticed that the reported increase in in-hospital mortality among obese pediatric trauma patients was not fracture-specific and likely reflects systemic injury severity rather than orthopedic complications alone.

### Biomechanical and biological mechanisms

The connection between obesity and impaired bone health involves intertwined mechanical and metabolic pathways. Mechanically, greater body mass generates higher axial and shear forces during trauma, predisposing individuals to complete and physeal fractures [35, 36]. This risk is compounded by factors like gait instability, reduced balance, and slower protective reflexes, which impair the ability to break a fall effectively [37, 38].

Biologically, obesity-induced insulin resistance and a state of chronic low-grade inflammation disrupt normal bone remodeling processes [11–14, 35]. This dysregulation affects the RANKL–RANK–OPG axis, tilting the balance towards osteoclast activation and bone resorption [14–17]. Concurrently, hyperinsulinemia and excess leptin further inhibit osteoblast differentiation, hampering bone formation [12, 35]. Paradoxically, while bone mineral density (BMD) is often higher in obese children [39, 40], the underlying trabecular microarchitecture and cortical geometry are often compromised. This results in bones that are denser yet mechanically weaker, ultimately increasing fracture susceptibility [41–49].

### Clinical implications and prevention

From a clinical perspective, pediatric obesity should be recognized as a significant modifier of fracture risk, pattern, and outcome. During management, clinicians should anticipate difficulties with fracture reduction, cast molding, and imaging interpretation due to thicker soft-tissue envelopes [35]. Surgical planning must account for the potential need for more rigid fixation constructs, longer instrumentation, and meticulous pin placement. Postoperatively, obese children warrant closer monitoring to detect early signs of displacement or loss of alignment.

On a preventive level, early lifestyle interventions focused on weight control, increased physical activity, and balanced nutrition could help reduce fracture incidence and promote better healing [50–52]. Screening for and managing obesity-related comorbidities, such as vitamin D deficiency and metabolic syndrome, is also advisable to mitigate underlying bone fragility and support optimal recovery.

Practical Surgical Considerations in Obese Pediatric Patients.

1. Lower threshold for operative fixation in forearm fractures.
2. Consider open reduction in lateral condyle fractures.
3. Avoid ESIN in femur/tibia > 50 kg → use rigid nails or plating.
4. Anticipate need for external fixation in unstable supracondylar fractures [53].
5. Close follow-up due to loss of reduction risk.

**Limitations of Current Evidence**

The interpretation of our findings must consider several limitations inherent in the available literature. Most included studies were retrospective (providing Level III or IV evidence) and utilized heterogeneous definitions for both BMI categories and fracture classifications. Important confounding factors—including physical activity levels, socioeconomic status, and ethnicity—were often not controlled for [33, 34]. Long-term follow-up data was scarce, and outcome measures were inconsistently reported, preventing a quantitative meta-analysis. Furthermore, the geographical representation is skewed, with a dominance of North American and European data, which limits the external validity of findings for other populations. To date, no randomized controlled trials have specifically investigated obesity as a primary modifier of fracture outcomes in pediatric patients.

## Future research directions

Future research should prioritize prospective, multicenter cohort studies that employ standardized BMI thresholds and consistent outcome definitions. Stratified analyses based on age, sex, and pubertal stage are essential to clarify how developmental stage influences the bone’s response to mechanical load. Integrating advanced imaging modalities (such as DXA, pQCT, and MRI) could help quantify differences in bone geometry and quality across BMI categories. Additionally, studies combining biochemical analysis with biomechanical modeling are needed to elucidate how metabolic dysregulation interacts with structural bone weakness. Finally, interventional trials examining the effects of weight reduction, physical conditioning, and nutritional optimization are crucial for establishing evidence-based strategies to prevent fractures and improve postoperative recovery in obese children [51, 52].

## Conclusion

Moderate-quality evidence from observational studies supports a clear association between childhood obesity and an increased risk and complexity of long bone fractures, particularly in the lower extremities. Obese children are more prone to complete and physeal fractures, encounter greater technical difficulties during fracture fixation, and experience more postoperative complications. These findings underscore that pediatric obesity is not merely a comorbidity but a significant biomechanical and metabolic modifier of fracture behavior.

Clinically, surgeons should anticipate challenges in both nonoperative and operative settings, employ robust fixation strategies, and ensure diligent radiographic follow-up. Preventive counseling regarding weight management, nutrition, and physical activity is essential to reduce fracture risk and improve recovery outcomes.

However, the current evidence base is constrained by retrospective study designs and heterogeneous reporting. Well-designed, prospective multicenter studies that incorporate standardized BMI definitions, biomechanical assessments, and long-term follow-up are necessary to clarify causal mechanisms and optimize management protocols for obese pediatric patients with fractures.

## Data Availability

No datasets were generated or analysed during the current study.
